# Insight into the mechanical, thermodynamics and superconductor properties of NbRuB via first-principles calculation

**DOI:** 10.1038/srep19055

**Published:** 2016-01-12

**Authors:** Wenyan Tian, Haichuan Chen

**Affiliations:** 1 College of Electronics and Information Engineering, Taiyuan University of Science and Technology, Taiyuan 030024, China; 2 College of Electrical Engineering and Information Technology, Xihua University, Chengdu 610039, PR China

## Abstract

Using the first-principles calculations, the electronic structure, chemical bonding, mechanical, thermodynamics and superconductor properties of NbRuB are investigated. The optimized lattice parameters were in good agreement with the experimental data. The analysis of the density of states and chemical bonding implies that the metallic behavior of NbRuB originates from the Ru and Nb, and the bonding behaviors are a mixture of covalent-ionic bonds. The bulk modulus, shear modulus, Young’s modulus, Poisson’s ratio and hardness of NbRuB were calculated. The results reveal that the NbRuB is ductility and the Vickers hardness is 15.06 GPa. Moreover, the 3D dependences of reciprocals of Young’s modulus is also calculated and discussed, showing strong anisotropic character for NbRuB. Finally, the Debye temperature and superconducting transition temperature are obtained.

Transition-metals borides (TMB) belong to a fascinating class of materials possess interesting physical and chemical properties. Almost both binary and ternary TMB have high hardness and ultraincompressible properties such as RuB_2_ and Ta_2_OsB_2_^1^,^2^. Nb-Ru-B belongs to the ternary TMB, with excellent mechanical and thermodynamic properties, and can be used to design electron device, *etc*. Up to now, four kinds of Nb-Ru-B compounds had been reported: Nb_7_Ru_6_B_8_, Nb_3_Ru_5_B_2_, Nb_2_RuB_2 _and NbRuB^3^,^4^,^5^,^6^,^7^. Infact, to the best of our knowledge, Nb_2_RuB_2_ has not been synthesized yet and its crystal structure is unknown.

Zheng *et al.*^6^ prepared new ternary borides Nb_7_Ru_6_B_8_ by arc-melting the foils of niobium (Nb), ruthenium (Ru) and crystalline boron (B) on a water-cooled copper hearth under argon. The crystal structures of Nb_7_Ru_6_B_8_ belong to the large group of the derivatives of the AlB_2_ structure type. The electronic density of states of Nb_7_Ru_6_B_8_ exhibits metallic character with a pseudogap below the Fermi level (*E*_*f*_). Hermus *et al.*^3^ successfully synthesized polycrystalline powders as well as single crystals of Nb_3_Ru_5_B_2_ by arc-melting the elements in a water-cooled copper crucible in an argon atmosphere using a tungsten tip as a second electrode and characterized by energy-dispersive X-ray and X-ray diffraction methods. It is the ternary phase of the type A_3_T_5_B_2_ adopting the Ti_3_Co_5_B_2_ structure type and containing a group eight transition metal at the T sites. They predicted the Nb_3_Ru_5_B_2_ is a metallic conductor with a deep pseudogap around the *E*_*f*_. Touzani *et al.*^5^ predicted “Nb_2_RuB_2_” (with Nb_2_OsB_2_-type structure), and studied the chemical bond, electronic structure, magnetism and elastic properties by using the density functional theory. They find that the Nb_2_RuB_2_ is non-magnetic and ultraincompressible. But, the Nb_2_RuB_2_ has not been synthesized yet and thus its crystal structure is unknown. Mbarki *et al.*^4^ attempted to synthesize the unknown “Nb_2_RuB_2_”. However, their experimental work didn’t found the “Nb_2_RuB_2_”, but found new ternary transition metal borides “NbRuB”. The NbRuB crystallizes in the space group *Pmma* with a new structure type that consists of two layers: one contains Nb atoms and isolated B atoms, whereas the other contains Ru atoms and B2 dumbbells. Xie *et al.*^7^ reported the temperature dependent electrical resistivity, magnetic susceptibility, heat capacity and thermodynamic characterization of the superconducting transition by theory and experiment methods.

In the present work, the electronic structure, chemical bonding, elastic, hardness, Debye temperature and superconductor properties of NbRuB are investigated using the Vanderbilt-type ultrasoft pseudopotential plane-wave method in order to provide more detailed physical properties for the theorists and experimentalists for future theoretical and experimental work on this compound.

## Calculation methods

All theory calculations were performed using the CASTEP code^8^. The B: 2s^2^2p1, Ru: 4s^2^4p^6^4d^7^5s^1^ and Nb: 4s^2^4p^6^4d^4^5s^1^ electrons were treated as valence electrons. The generalized gradient approximation with the Perdew-Burke-Ernzerhof for solids (GGA-PBEsol)^9^ functional for the exchange-correlation was employed. A plane-wave basis with a cutoff energy of 600 eV was used to expand the wave functions. The *k*-point samplings in the Brillouin zone are 4 × 12 × 7 based on the Monkhorst-Pack method. The structural optimizations were determined using the Brodyden-Fletcher-Goldfarb-Shanno (BFGS)^10^ method.

## Results and Discussions

### Electronic Structure and Chemical Bonding

**Figure 1:**
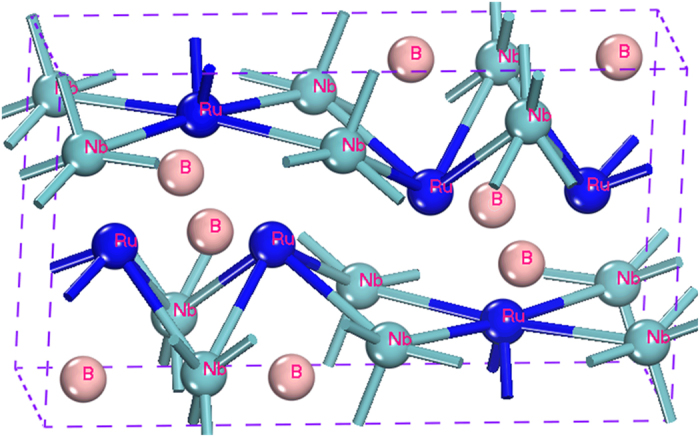
The crystal structure of NbRuB.

NbRuB has an orthorhombic structure with space group *Pmma*, as shown in Fig. 1. It is characterized by two different layers alternately stacked: The first layer contains Nb and B atoms, whereas the second layer is filled with Ru atoms and the B2 dumbbells. In NbRuB, there are two types of B atoms: one is in the center of a triangular prism with Ru at the six vertices, and the other exists as a B-B dimer inside a double-triangular prism of Nb. The optimized lattice parameters *a* = 10.833 Å, *b* = 3.141 Å and *c* = 6.324 Å are in well agreement with the experimental data reported in ref. 4 and ref. 7 (Table 1). There is a slightly underestimate of only 0.5% from the experimental data, which is due to the thermal expansion effects.

**Figure 2:**
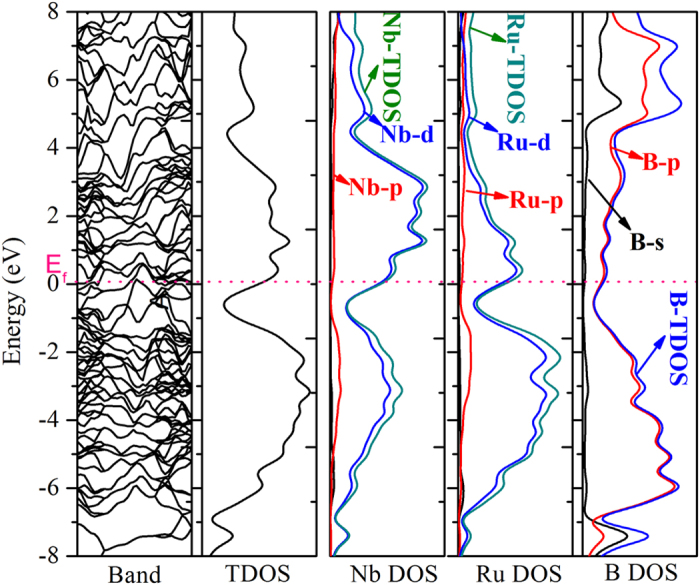
Band structure and density of states of NbRuB.

To gain further insights into NbRuB, the band structure, total density of states (TDOS) and partial density of states (PDOS) of Nb, Ru and B are plotted in Fig. 2. From −8 eV to the *E*_*f*_, the majority of the DOS stems from 4*d* states of Ru and Nb, followed by B-2*p* states. The conduction bands above the *E*_*f*_ originate mostly from Nb–4*d* and B-2*p* states, and small contributions from *s* and *p* states of Ru, Nb and B. From Fig. 2, the NbRuB exhibit metallic behavior because there is no band gap at the *E*_*f*_. Though electronic structure analysis, the metallic behavior of NbRuB originates from Nb and Ru metals which contribute nearly equally at the *E*_*f*_ of the DOS. The metallic behavior of NbRuB indicates that this material might be a superconductor and we will discuss it in the following.

Population analysis provides more insightful information on the chemical bonding characteristics. A high value of the bond population indicates a covalent bond, while a low value indicates an ionic interaction. In this work, the Mulliken method is used to calculate the overlap population and the charge, and the calculated results and the experiment data are listed in Table 2. From the Table 2, we can see that B and Ru atoms carry the negative charges and the positive charges are carried by Nb atom. The transferred charge from Nb to B and Ru are equal to 0.51*e*, 0.41*e*, 0.23*e* and 0.3*e*, respectively. The obtained transferred charge values suggest an effective valence state of Nb^0.75^Nb^0.7^Ru^−0.23^Ru^−0.3^B^−0.51^B^−0.41^.

Mulliken’s bond overlap population for nearest neighboring atoms is a measure of spatial charge density between bonding atoms, and this quantity does not totally depend on the valence charge transfer along the bond axis. The bond overlap populations can reflect the trend of bond ionicity. The ionicity of a bond based on bond overlap population can be calculated as follows^11^:


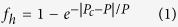


Where 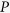 is the overlap population of bond, 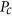 is the overlap population of the bond in a pure covalent crystal (We assume the 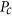 = 1 for purely covalent bond). The 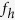 = 0 for a purely covalent bond, while 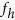 = 1 indicates a purely ionic bond. The bond length, population and population ionicity of the NbRuB are given in Table 2. From Table 2, it is clear that the strongest chemical interaction in NbRuB is the B1-B1 dumbbell, and the B-Ru shows a high level of covalency and a low level of ionicity, while the Ru-Ru, Nb-Ru and B-Nb shows almost a complete ionicity (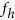
≈1). Thus, we conclude that the bonding behaviors of NbRuB may be described as a mixture of covalent-ionic bonds.

### Mechanical properties

The elastic constants not only provide a link between the mechanical and dynamical behavior of crystals, but also provide important information concerning the nature of the forces operating on solids. The elastic constants of NbRuB by the strain-stress method are listed in Table 3. Since the elastic constants 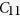, 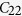 and 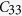 can be directly related to the crystallographic *x*, *y*, and *z* axes, respectively. As seen in Table 3, the observed ordering of elastic constants is 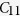 ≈ 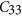 > 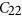, which represents a relative weakness of lattice interactions along the crystallographic *y* axis. Moreover, The Young’s modulus for the different directions have also been determined (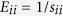, *i* = 1, 2 or 3, where 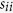
are the diagonal elements of the compliance matrix). The calculated Young’s modulus for the different directions are 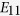 = 392.8 GPa, 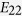 = 188.8 GPa and 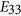 = 319.0 GPa, respectively. Finally, the 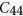, 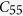, and 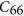 indicate the shear elasticity applied to the two-dimensional rectangular lattice in the (100), (010) and (001) planes. In Table 3, 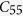 is smaller than 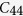 and 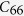, indicating the soft shear transformation along the (010) plane.

To be mechanically stable, the elastic constant should satisfy the generalized elastic stability criteria. For the NbRuB crystals, the stability criteria are given as follows^12^:


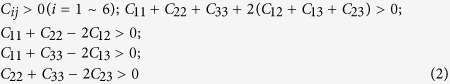


Clearly, the calculation results suggest that NbRuB is mechanically stable at the ambient condition. According to the calculated elastic constants, the polycrystalline bulk modulus 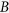 and shear modulus 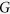 are obtained using the Voigt-Reuss-Hill (VRH) approximation. Once bulk modulus 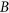 and shear modulus 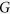 are obtained, the Young’s modulus *E* and Poisson’s ratio 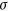 can be computed^13^. The calculated values of the 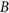, 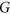, *E* and 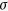 of NbRuB are also listed in Table 3. The *B* is a measure of the resistance against volume change imposed by the applied pressure, while the *G* denotes the resistance against the reversible deformations upon shear stress. The calculated *B* and 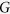
of NbRuB is 293 GPa and 156 GPa, respectively, larger than that of Nb_2_RuB_2_ (*B* = 272 GPa, *G* = 146 GPa)^2^. Moreover, the shear modulus *G* of NbRuB is below 200 GPa, indicating the relative low resistance to shape change at a constant volume. The high bulk and shear modulus of NbRuB maybe be derived from the strong covalent bonding. According to the Pugh criterion^14^, the NbRuB is ductile material because its *B/G* value is higher than 1.75. Indeed ductile materials are generally metallic even though some metals can be brittle. In a word, the large elastic modulus and low Poisson’s ratio show that the NbRuB would be potential hard materials.

The Vickers hardness (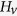), the intrinsic resistance to deformation when a force is applied, is another interesting property of materials. The Vickers hardness 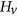
of complex crystals should be calculated by a geometric average of all bonds^15^:


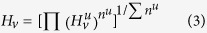



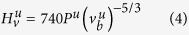



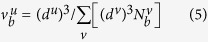


where 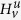 is the hardness of binary compound composed by 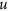-type bond, 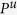 is the Mulliken overlap population of the 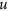-type bond, 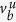 is the volume of a bond of type 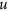, 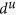 is the bond length of type 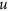, and 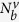 is the bond number of type 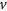
per unit volume. The calculated volume, bond parameter, and hardness of NbRuB crystals are presented in Table 4. It is found that the Vickers hardness value of polycrystalline NbRuB is 15.80 GPa, which is slight bigger than that of RuB_1.1_ (14.5 GPa). The higher level of hardness of the NbRuB is attributed to its faint metallicity, wherein the PDOS of NbRuB at the Fermi level is much smaller than that of RuB_1.1_. As well known, the hardness is related to the elastic and plastic properties of the material. The Vickers hardness of NbRuB is also can be estimated by the Chen’s empirical model. The calculated Vickers hardness of NbRuB from Chen’s empirical model is listed in Table 4. It is found that the Vickers hardness is 15.29 GPa, which is close to 15.80 GPa based on expression (3–5) indicate that NbRuB is hard materials (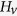 > 10 GPa).

### Elastic anisotropy

**Figure 3:**
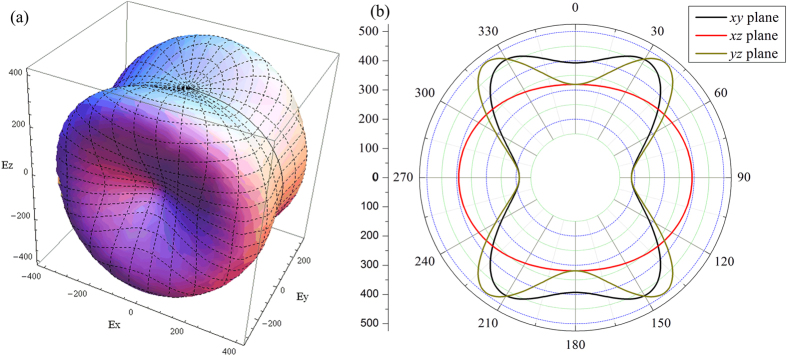
3D directional dependence of the Young’s modulus (in GPa) and its projection on the *xy*, *xz* and *yz* planes of NbRuB.

Elastic anisotropy is an important physical property of materials and plays a vital role in technological and industrial applications. To better describe the features of elastic anisotropy, the three-dimensional (3D) dependences of reciprocals of Young’s modulus can be obtained by the following equation^17^:


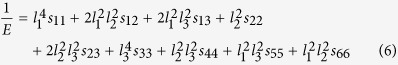


where 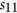, 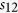 and 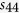 are the elastic compliances, 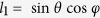, 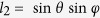 and 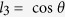 are the directional cosines of angles with the three principal directions. For a perfectly isotropic material, the 3D curved surface exhibits a spherical shape, while the deviation degree from the spherical shape indicates the anisotropic character of the crystal. The obtained 3D curved surface for NbRuB is shown in Fig. 3 (a). It demonstrates that the NbRuB exhibit a strong anisotropic character in Young’s modulus. The projections on the *xy*, *xz* and *yz* planes show more details regarding the anisotropic properties of the Young’s modulus. The 2D projections of Young’s modulus in those planes are shown in Fig. 3 (b). From the Fig. 3 (b), we can see that the *E*_min_ = 188.8 GPa (188.8 and 319.0 GPa) and *E*_max_ = 466.6 GPa (495.8 and 392.8 GPa), the ratio *E*_max_/*E*_min_ = 2.47 (2.63 and 1.23) in the *xy*, *yz* and *xz* planes, respectively, indicating the Young’s modulus of the *xz* plane has a weak anisotropic character compared to the other planes.

To further investigate the anisotropy, we calculated the universal anisotropic index 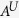
18. For an isotropic material, 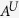 is equal to zero, the deviation of 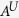
from zero indicates the presence of elastic anisotropy. The calculated 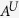 is collected in Table 3. It is noted that the NbRuB is significantly anisotropic.

### Thermodynamic properties

The Debye temperature is the temperature of the highest normal mode of vibration in a crystal, and it provides insight into the thermodynamics of the material. However, the Debye temperature (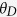) is not a strictly determined parameter, various estimates may be obtained through well established empirical or semi-empirical formula. In this work, we calculated the Debye temperature through elastic constants base on the Anderson’s equation^19^. The calculated values of the Debye temperature are presented in Table 3. The Debye temperature 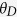 is determined to be 587.6K, which is larger than that (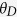 = 468 K)^7^ obtained from fitting the capacity to the Debye model in the low-temperature limit. These differences are not unexpected since the values of the Debye temperatures obtained from different definitions/experiments are expected to be different although close.

From the value of DOS at *E*_*f*_ obtained by theory calculate and experiment measure, we have estimated the value of electron-phonon coupling constant 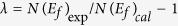
20. The superconducting transition temperature 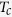 has been estimated using the McMillan formula^21^: 
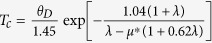
. Where 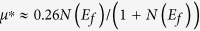 is the Coulomb pseudopotential, which characterizes the strength of the electron-electron Coulomb repulsion^22^. The calculated values of 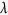, 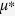
and 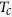
are given in Table 3 along with the experimental data. In our work, the calculated 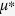 = 0.16, 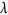 = 0.584, the estimated 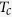 = 4.5 K, which is larger than the experimental data 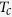 = 3.1 K. It should be considered that the phonon calculation was not performed with a shifted set of bands, and given that the effect of that shift was to decrease *N*(*E*_*f*_). As seen from Fig. 2, the contribution of the 4*d*-state is larger than those of the 5*s* and 4*p* states. The larger contribution of 4*d* state electrons clearly shows the possibility of superconductivity in NbRuB.

## Conclusions

In summary, the electronic structure, chemical bonding, mechanical and thermodynamic properties of NbRuB have been studied via first-principles. The optimized lattices parameters are slightly underestimate the experimental data. The DOS reveals that NbRuB exhibit metallic behavior, and the valence bands stems from 4*d* states of Ru and Nb, the conduction bands originate mostly from Nb-4*d* and B-2*p* states. The Mulliken charge population shows this compound has mixed covalent-ionic property. The elastic properties of this material are analyzed, and the results show that NbRuB is mechanically stable and an elastic anisotropy. The calculated *B/G* ratio and Vickers hardness shows that NbRuB is ductile and hard material. Moreover, the values of the superconducting temperature 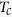 of NbRuB is predicted to be 4.5 K, which is larger than the experimental value 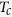 = 3.1 K. The superconductivity of NbRuB may be related to its electronic properties and the geometry structure.

## Additional Information

**How to cite this article**: Tian, W. and Chen, H. Insight into the mechanical, thermodynamics and superconductor properties of NbRuB via first-principles calculation. *Sci. Rep.* **6**, 19055; doi: 10.1038/srep19055 (2016).

## Figures and Tables

**Table 1:** The calculated lattice constants of NbRuB.

NbRuB *Pmma* (*No. 51*)					
	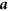 (Å)	*b* (Å)	*c* (Å)	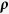 (g/cm)	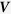 (Å^3^)
This calc.	10.833	3.141	6.324	9.484	215.14
Expt ref. 7	10.867	3.156	6.350	9.408	217.67
Ref. 4	10.869	3.165	6.350	9.339	218.47

**Table 2:** Mulliken population analysis results of NbRuB.

Bond	*s*	*p*	*d*	Total	Charge (e)
B1	1.03	2.48	0	3.51	−0.51
B2	1.02	2.39	0	3.41	−0.41
Nb1	2.06	6.34	3.85	12.25	0.75
Nb2	2.07	6.40	3.83	12.30	0.70
Ru1	2.34	6.87	7.02	16.23	−0.23
Ru2	2.36	6.93	7.01	16.30	−0.30
Bond	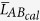 (Å)	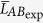 (Å)^4^	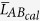 (Å)^4^	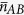 (*e*)	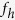
B1-B1	1.916	1.88	1.93	0.6	0.49
B1-Nb	2.362–2.465	2.371–2.493	2.39–2.5	0.33–0.38	0.80–0.86
B2-Nb	2.565/2.654	2.56/2.675	2.60/2.69	−0.01/0.04	1
B1-Ru	2.409/2.432	2.41/2.46	2.44/2.47	0.22/0.24	0.96/0.97
B2-Ru	2.211/2.217	2.223/2.25	2.24/2.25	0.74	0.30
Nb1-Ru	2.732/2.849	2.745/2.863	2.77/2.89	−0.13/0.07	≈1
Nb2-Ru	2.698/2.741	2.704/2.755	2.73/2.78	−0.16/−0.08	…
Nb-Nb	2.979	2.989/3.165	3.01–3.18	−0.38	…
Ru-Ru	2.70–2.862	2.713–2.989	2.73–2.78	−0.3/0.04	1

**Table 3:** Calculated elastic constants 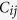, elastic compliance constants 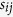
(×10^−3^), polycrystalline elastic modulus, elastic anisotropy, Debye temperature and superconducting properties for NbRuB.

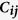 (GPa)	*C*_11_	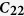	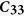	*C*_44_	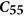	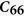	*C*_12_	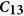	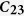
	523.2	361.9	504.8	212.6	181.8	196.2	217.2	154.9	259.3
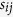 (1/GPa)	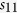	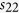	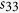	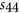	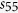	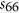	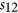	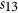	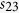
	2.5458	5.2959	3.1350	4.7047	5.4991	5.0974	−1.5322	0.0060	−2.2504
***bulk modulus*** 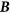 **(GPa)**			***shear modulus G*** **(GPa)**			*Young’s modulus* 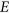 (GPa)	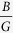	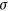	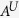
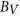	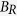	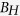	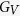	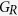	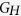				
297.7	292.1	293.4	168.7	143.0	155.8	397.2	1.88	0.27	0.91
	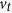 (m/s)	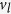 (m/s)	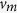 (m/s)	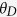 (K)		N(E_f_) states/eV	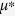	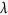	*T*_*c*_(K)
Cal.	4053.5	7269.7	4513.3	587.6	Cal.	1.73	0.16	0.584	4.5
Ref. 7				468	Ref. 7	2.74	0.15	0.544	3.1

**Table 4:** Calculated bond parameters and Vickers hardness for NbRuB.

Bond	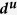 (Å)	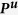	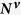	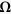 (Å^3^)	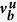	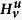 (GPa)	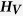 (GPa)	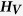 (GPa)
B1-B1	1.916	0.6	2	215.144	1.703	182.780	15.80	15.29
B2-Ru	2.211	0.74	4		2.620	109.954		
B2-Ru	2.217	0.74	2		2.620	109.954		
B1-Nb	2.362	0.36	4		3.191	38.514		
B1-Nb	2.372	0.33	4		3.234	34.527		
B1-Ru	2.409	0.24	4		3.389	23.228		
B1-Ru	2.432	0.22	4		3.486	20.314		
B1-Nb	2.465	0.38	4		3.627	32.836		
B2-Nb	2.565	0.04	2		4.089	2.831		
B2-Nb	2.654	−0.01	4		4.532	−0.596		
Nb2-Ru	2.698	−0.16	2		4.758	−8.798		
Ru-Ru	2.699	−0.3	6		4.767	−16.443		
Nb1-Ru	2.732	−0.13	4		4.940	−6.714		
Nb2-Ru	2.741	−0.08	4		4.990	−4.063		
Ru-Ru	2.862	0.04	2		5.678	1.638		
Nb-Nb	2.979	−0.38	2		6.405	−12.728		
Nb-Ru	2.738	0.07	4		4.551	4.145		
